# Abdominal skin inflammation as an initial symptom of a perforating gastric foreign body: A case report

**DOI:** 10.1097/MD.0000000000022534

**Published:** 2020-10-02

**Authors:** Lili Zhang, Lifang Liu, Jiangbo Shao, Fangfang Sun, Lirong Zhao

**Affiliations:** Department of Ultrasound Diagnosis, The First Hospital of Jilin University, China.

**Keywords:** elderly, foreign body ingestion, perforation, small curvature, stomach

## Abstract

**Rationale:**

Foreign bodies are frequently ingested, but only approximately 1% of them cause perforation. Perforations in the lesser curvature of the stomach are exceedingly rare. Here, we report a case of gastric perforation in the lesser curvature caused by a foreign body. The patient presented to the clinic complaining of abdominal skin swelling and reddening with upper abdominal discomfort as the initial symptoms.

**Patient concerns:**

An 83-year-old female presented with a mass in the middle of the epigastrium for 10 days. Physical examination found an apparent local tenderness and inflammatory mass in the upper abdominal wall. Her body temperature was normal (37.5°C) and the white blood cell count was elevated (8.12 × 10^9^/L [reference value 3.5–9.5 × 10^9^/L]).

**Diagnoses:**

The ultrasound examination of the abdomen revealed a 4 cm strip-like hyperechoic object entangled in the muscles of the abdominal wall. The computed tomography scan revealed a thin strip of bone-like hyperdense shadow. Intraoperative findings showed a sharp fishbone protruding from the lesser curvature of the stomach into the abdominal cavity, part of which remained in the gastric cavity. The postoperative pathological report revealed chronic suppurative inflammation with abscess and sinus canal formation.

**Interventions & outcomes:**

The patient underwent a gastric foreign body removal with partial gastrectomy. Anti-inflammatory treatment post-surgery rapidly relieved the patient's symptoms of discomfort in the upper abdomen. At the 1-month follow-up, the patient showed no discomfort in the upper abdomen and the inflammatory mass was no longer present.

**Lessons:**

A foreign body had penetrated through the lesser curvature of the stomach, an area with a flat gastric wall, which occurs infrequently. In such cases, computed tomography is the gold standard for diagnosis of foreign bodies in the digestive tract. Ultrasound can also be used as a supplemental diagnostic technique. It is recommended that people who wear dentures should exercise caution while eating, especially when the food contains bones.

## 1 Introduction

The ingestion of foreign bodies is a frequent occurrence, although most foreign bodies manage to pass through the gastrointestinal tract without causing any complications, such as an obstruction. However, approximately 1% of ingested foreign bodies, especially in the case of large, sharp, or pointed objects may cause perforations and create significant risk.^[1]^ Perforations usually occur in areas with acute angles or physiological narrowing^[2]^ such as the gastroesophageal junction or pylorus. However, perforations in the lesser curvature of the stomach are very rare. Here, we report a case of gastric perforation caused by a foreign body in the lesser curvature of the stomach that presented to the clinic with an initial symptom of abdominal skin inflammation.

## 2 Case report

### 2.1 Patient information

An 83-year-old female was referred to our hospital with a mass in the middle of the epigastrium for 10 days. The patient also complained of epigastric discomfort for the past 6 months. Her symptoms did not resolve even after taking some medicine prescribed in the clinic. She did not have a history of gastropathy and affirmed that she had been wearing dentures for many years.

### 2.2 Clinical findings

Physical examinations found that the skin of the upper abdomen was red and swollen, and there was an apparent local tenderness with an inflammatory mass. The size of the skin protrusion measured approximately 4 × 5 cm, and there was no evidence of a surrounding scar or ulcer. There was no rebound pain or muscle tension in the remaining area of the abdomen. The patient had an average body temperature of 37.5°C, with an increased white blood cell count (18.12 x× 10^9^/L [reference value 3.5–9.5 × 10^9^/L]) on laboratory examination.

### 2.3 Diagnostic assessment

**Figure 1:**
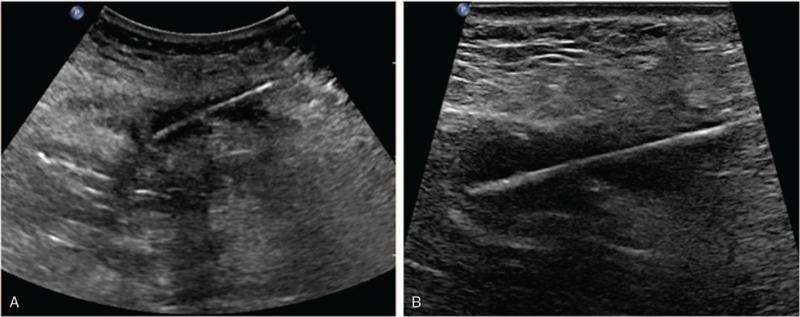
Ultrasound (US) examination of the abdomen. The US examination revealed a 4 cm strip-type hyperechoic object in the muscle of the abdominal wall.

An ultrasound (US) examination of the abdomen revealed a 4 cm strip-like hyperechoic object entangled in the muscles of the abdominal wall, which was 2.5 cm away from the surface of the skin. The hyperechoic object penetrated the muscle layer and was partially located in the abdominal cavity, while the surrounding soft tissues were also hypoechoic (Fig. 1). In the US finding, the atypical stripe echo in the abdominal wall was considered a foreign body.

**Figure 2:**
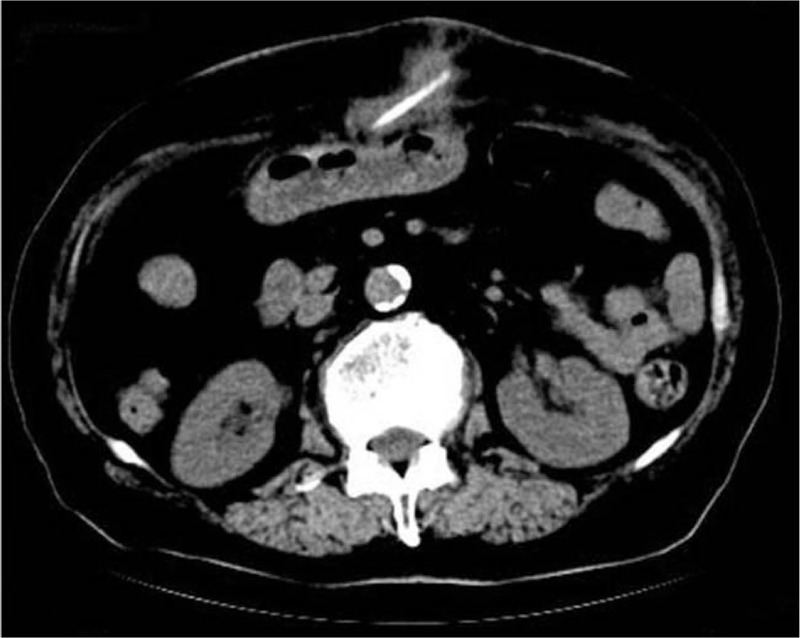
Computed tomography (CT) of the abdomen. The CT scan showed a flaky, soft tissue density shadow between the anterior abdominal wall and subcutaneous fat, and a thin strip of bone-like hyperdense shadow.

A computed tomography (CT) scan revealed a flaky, soft tissue density shadow between the anterior abdominal wall and subcutaneous fat, and a thin strip of bone-like hyperdense shadow. The fatty space in the lesser curvature of the anterior abdominal wall was turbid, with continually increasing density and high-density shadows. The CT results confirmed the presence of a foreign body entangled between the abdominal cavity and the abdominal wall that was causing infection (Fig. 2).

### 2.4 Treatment and follow-up

**Figure 3:**
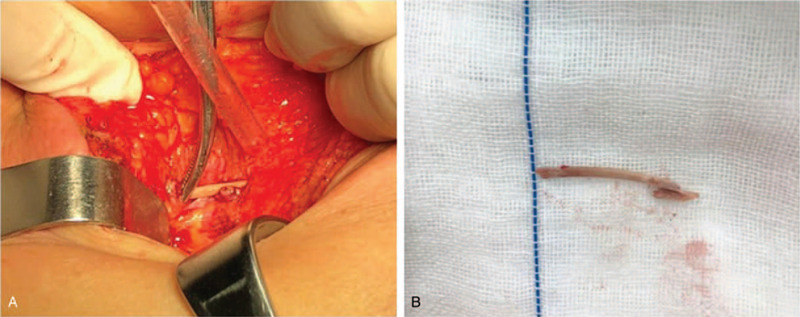
Surgical findings. Intraoperative findings revealed a sharp fishbone protruding from the lesser curvature of the stomach into the abdominal cavity, part of which remained in the gastric cavity.

**Figure 4:**
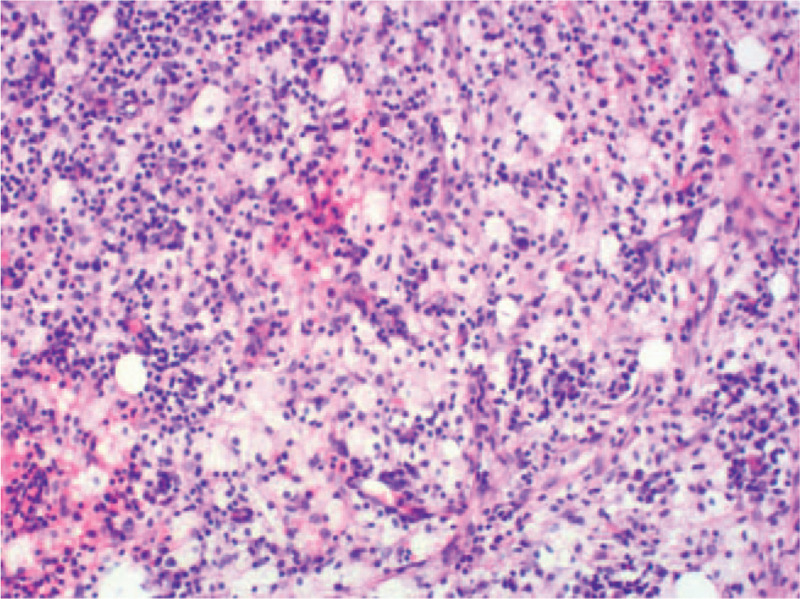
Histopathological examination. High-powered microscopy showed numerous lymphocytes and plasma cells, with a few eosinophilic granulocytes and foamy cells (original magnification 200×).

The patient underwent a gastric foreign body removal by partial gastrectomy under general anesthesia. Intraoperative findings showed a sharp fishbone protruding from the lesser curvature of the stomach into the abdominal cavity, while a part of the bone remained in the gastric cavity (Fig. 3). The foreign body measuring approximately 4.2 cm was removed. The sinus tract in the gastric wall was cleared and closed. The postoperative pathology of the tissue from the gastric wall revealed chronic suppurative inflammation with abscess and sinus canal formation. The foreign body was surrounded by proliferating granular and fibrous tissues with local bleeding (Fig. 4). The patient received anti-inflammatory treatment following the surgery, and the symptoms of discomfort in the upper abdomen improved rapidly. The patient was discharged from the hospital 9 days after the surgery.

At the 1-month postoperative follow-up, the patient showed no discomfort in the upper abdomen, and the inflammatory mass of the abdominal wall was no longer present.

## 3 Discussion

Foreign body ingestion is common, and the majority (80%–90%) of these cases resolve without any complications when the object passes through the gastrointestinal tract.^[2]^ However, foreign bodies can rarely cause complications such as obstructions, perforations, bleeding, ulcers, peritonitis, and in extreme cases can even lead to death.^[3]^ Accidental ingestion of a foreign body most commonly occurs through the mouth or pharynx, followed by the gastrointestinal tract.^[4]^ Rarely, these objects migrate from the digestive tract to the pancreas,^[5]^ or the liver to cause liver abscess,^[6]^ or they penetrate the pharynx to the surface of the skin.^[4]^ The fishbone is one of the most common accidentally ingested dietary foreign bodies, accounting for up to 84% of all foreign bodies ingested accidentally.^[7]^ In this case, the foreign body was identified as a fishbone only after the intraoperative extraction.

Although children are more susceptible to foreign body ingestion, it is also commonly observed in the elderly population. In elderly, wearing dentures is an important risk factor for foreign body ingestion as it diminishes the tactile sensation of the palate. The palate offers protection by preventing ingestion of sharp or hard-textured objects in a food bolus with its sensory reflex.^[4]^ As such, in this case the patient had been wearing dentures for many years, which might have caused her to accidentally ingest a foreign body.

In this case, the foreign body penetrated through the lesser curvature of the stomach, which occurs infrequently due to the large surface area and flat gastric wall of this region. As reported by Karadeniz et al,^[8]^ potential lacunae in the gastric wall, such as Meckel's diverticulum, are responsible for easy embedding of a foreign body. However, the postoperative pathology of the gastric tissue from this patient showed only suppurative inflammation, and the discomfort of the upper abdomen improved rapidly following the operation, which ruled out gastric intraluminal abnormalities. In a similar case, Yoshioka et al^[9]^ proposed that foreign body penetration of the gastric wall after accidentally swallowing an object during dinner might be due to the excitement of the parasympathetic nervous system and the active peristaltic activity of the gastrointestinal tract at night.

The length of time between ingestion of the foreign body and the appearance of complications varies greatly and can range from a few hours to many years.^[10]^ This contributes to varying degrees of difficulties in the diagnosis of foreign body ingestion. Patients that have ingested nondietary foreign bodies usually have a clear history, and the diagnosis of these patients is often definite.^[11]^ On the contrary, patients with a history of dietary foreign body ingestion presenting with a broad-spectrum of nonspecific clinical symptoms make the investigation of dietary foreign body extremely difficult. The patient in this case did have a history of dietary foreign-body ingestion but was unable to recall the incident. We initially judged that the redness and swelling of the abdominal wall found at the beginning of the examination was localized soft tissue inflammation. The foreign body in the abdominal wall was not found until a routine US examination of the digestive system was performed. Various imaging techniques can be used for the diagnosis of gastrointestinal foreign bodies. Among these techniques, CT scans play an essential role,^[11]^ since they are able to unveil the location of foreign body, the association between the foreign bodies and the surrounding tissues, the local perforated area, and the overall condition of the abdominal cavity. An US examination can also be used to detect foreign bodies in the digestive tract because of its convenience and repeatability, and utilization as an analytical technique, especially when radiolucent foreign bodies are suspected and the results of CT and gastroscopy are negative.^[12]^ However, it has a limited application in the diagnosis of foreign bodies that are located deep in the body or blocked by intestinal cavity gas.

## 4 Conclusion

A foreign body had penetrated through the lesser curvature of the stomach, a region with a large surface area and flat gastric wall, which occurs infrequently. In such cases, CT is the gold standard for diagnosis of foreign bodies in the digestive tract. US can be used as a supplemental diagnostic technique. It is recommended that people who wear dentures should exercise caution while eating, especially when the food contains bones.

## Author contributions

Lili Zhang and Lifang Liu designed the study, conducted all searches, appraised all potential studies, and wrote and revised the draft manuscript and subsequent manuscripts. Lirong Zhao revised the draft manuscript and subsequent manuscripts. Jiangbo Shao and Fangfang Sun assisted with the presentation of findings and assisted with drafting and revising the manuscript. All authors read and approved the final manuscript.
